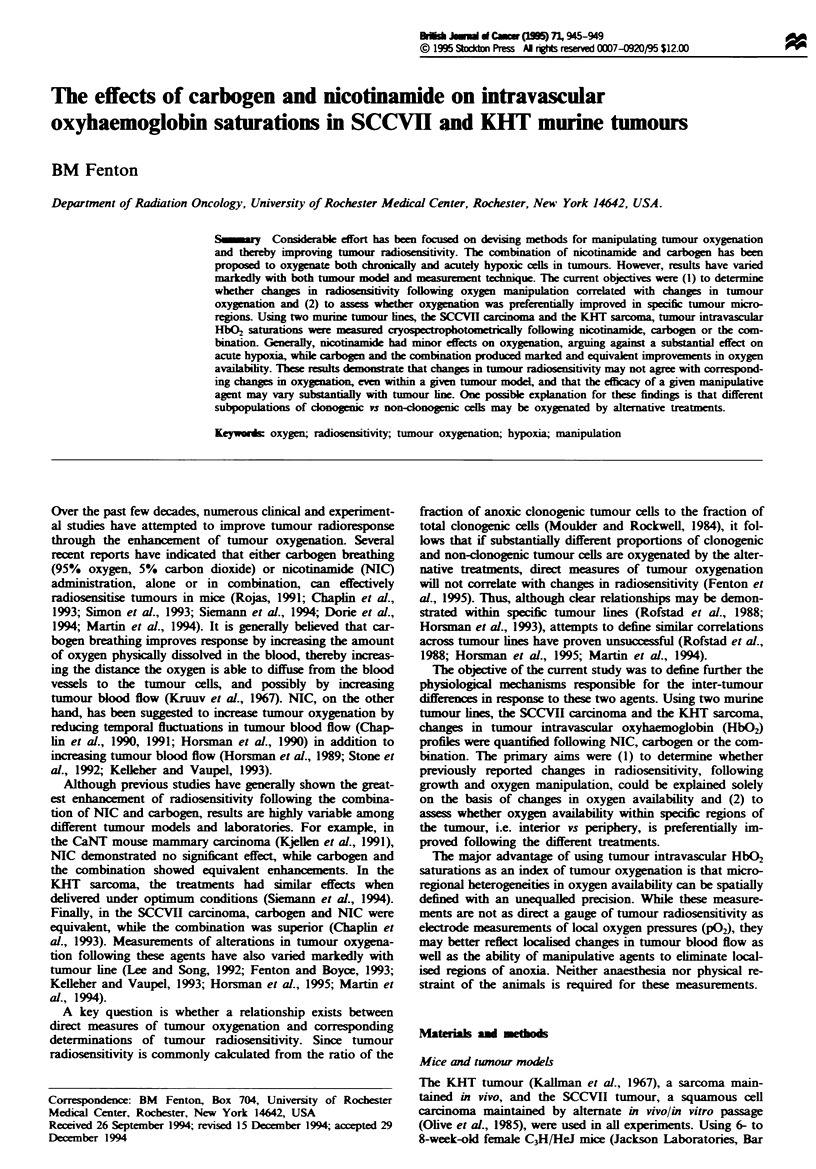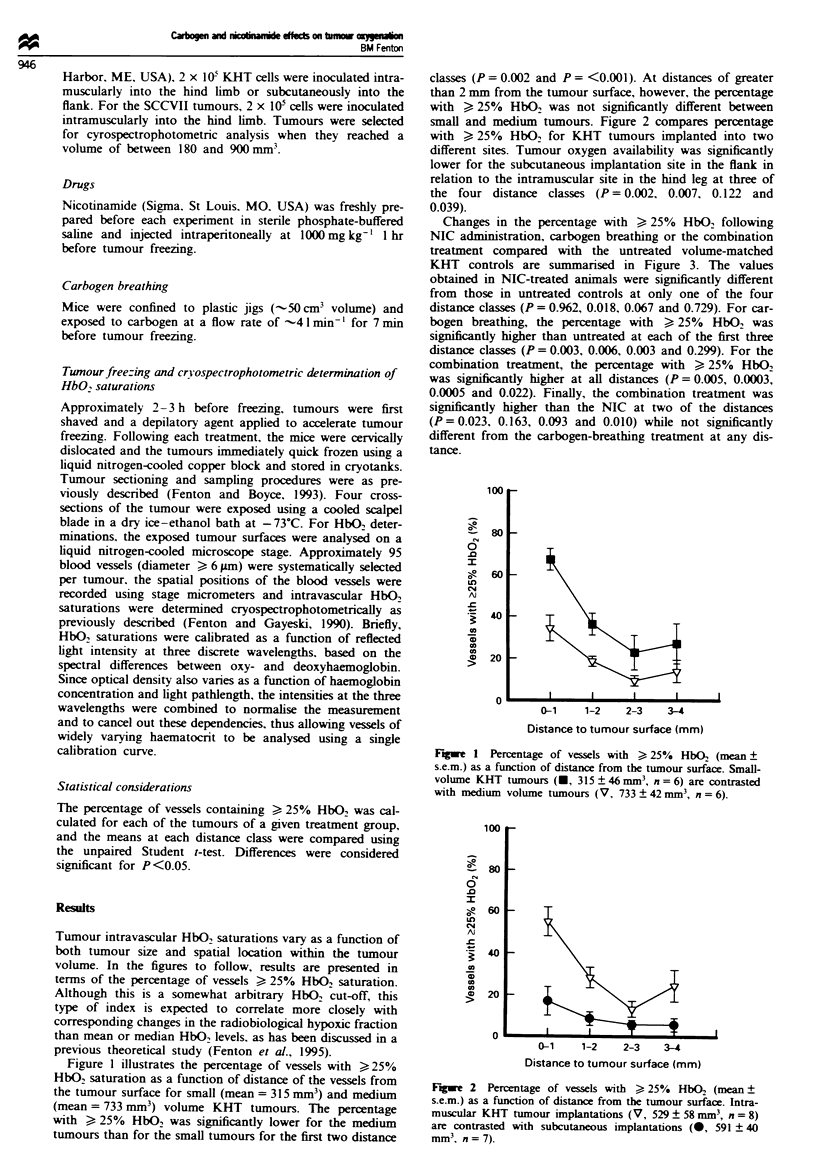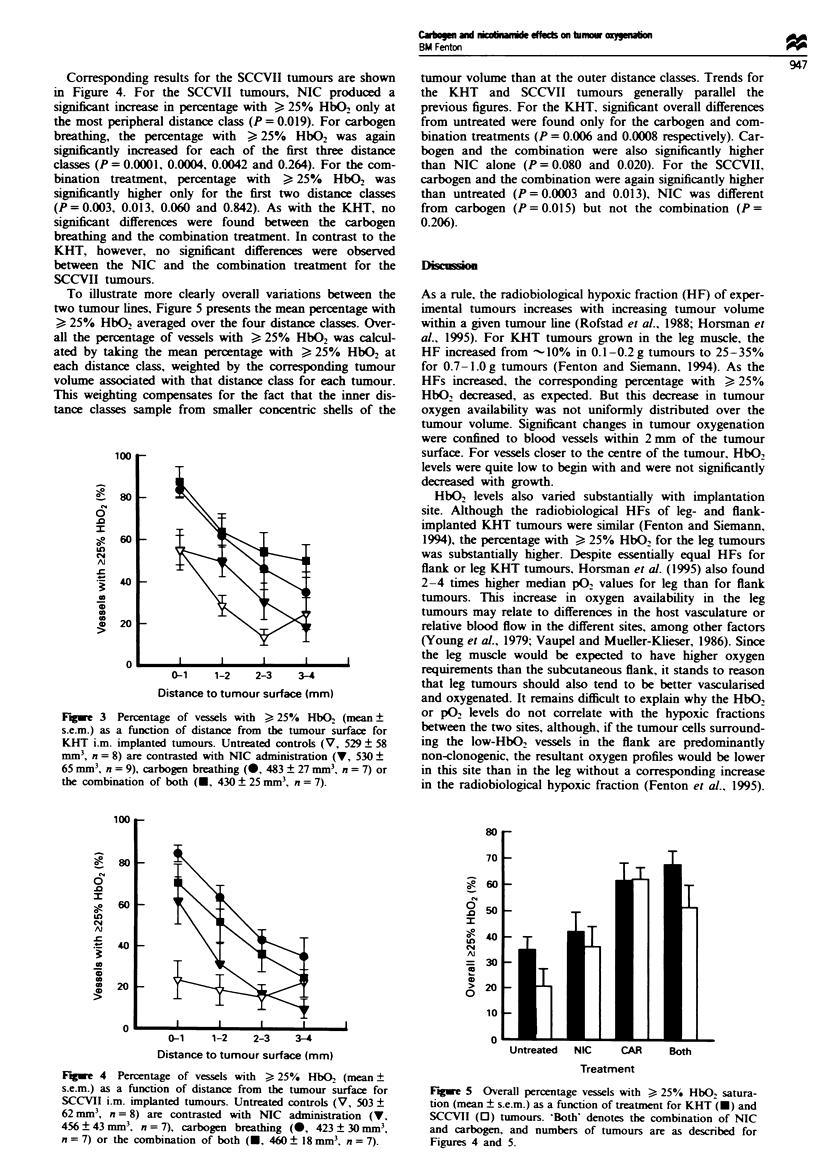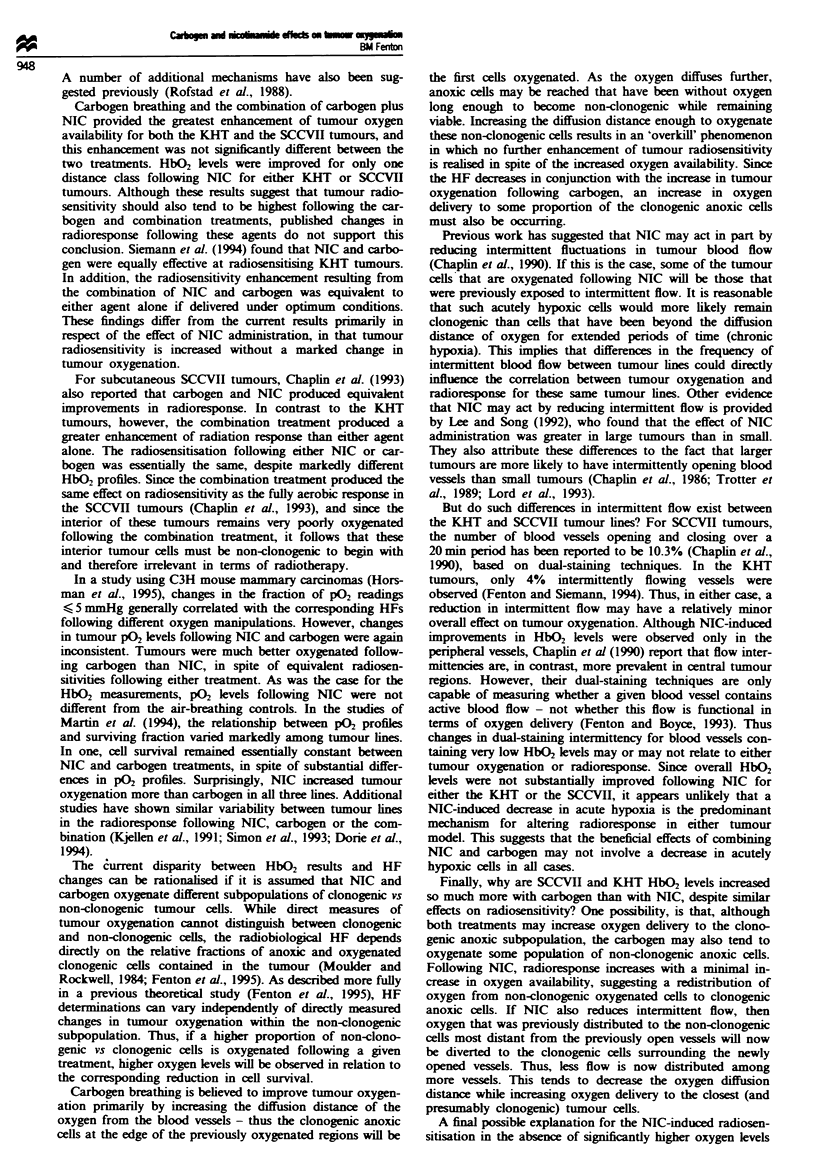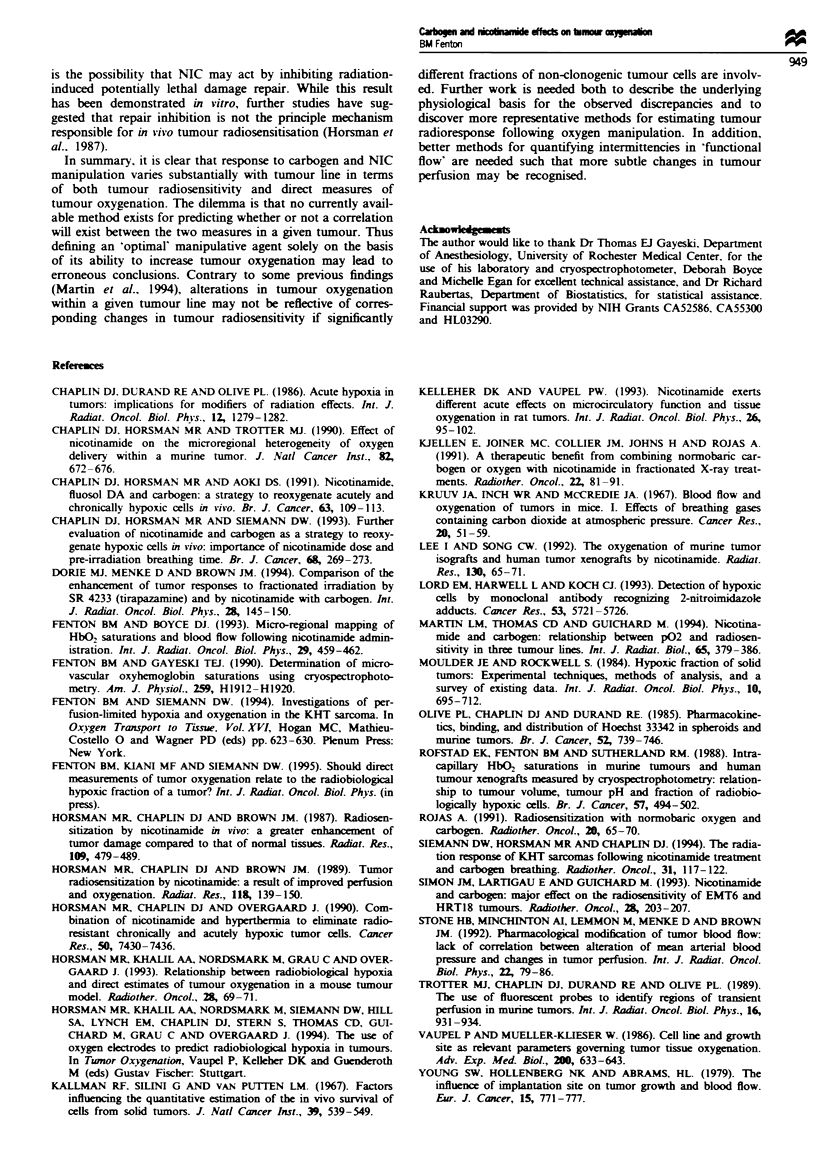# The effects of carbogen and nicotinamide on intravascular oxyhaemoglobin saturations in SCCVII and KHT murine tumours.

**DOI:** 10.1038/bjc.1995.183

**Published:** 1995-05

**Authors:** B. M. Fenton

**Affiliations:** Department of Radiation Oncology, University of Rochester Medical Center, New York 14642, USA.

## Abstract

Considerable effort has been focused on devising methods for manipulating tumour oxygenation and thereby improving tumour radiosensitivity. The combination of nicotinamide and carbogen has been proposed to oxygenate both chronically and acutely hypoxic cells in tumours. However, results have varied markedly with both tumour model and measurement technique. The current objectives were (1) to determine whether changes in radiosensitivity following oxygen manipulation correlated with changes in tumour oxygenation and (2) to assess whether oxygenation was preferentially improved in specific tumour micro-regions. Using two murine tumour lines, the SCCVII carcinoma and the KHT sarcoma, tumour intravascular HbO2 saturations were measured cryospectrophotometrically following nicotinamide, carbogen or the combination. Generally, nicotinamide had minor effects on oxygenation, arguing against a substantial effect on acute hypoxia, while carbogen and the combination produced marked and equivalent improvements in oxygen availability. These results demonstrate that changes in tumour radiosensitivity may not agree with corresponding changes in oxygenation, even within a given tumour model, and that the efficacy of a given manipulative agent may vary substantially with tumour line. One possible explanation for these findings is that different subpopulations of clonogenic vs non-clonogenic cells may be oxygenated by alternative treatments.


© 1995 Stockton Press All rights reserved 0007–0920/95 $12.00

### The effects of carbogen and nicotinamide on intravascular oxyhaemoglobin saturations in SCCVII and KHT murine tumours

BM Fenton

*Department of Radiation Oncology, University of Rochester Medical Center, Rochester, New York 14642, USA.*

**Summary** Considerable effort has been focused on devising methods for manipulating tumour oxygenation and thereby improving tumour radiosensitivity. The combination of nicotinamide and carbogen has been proposed to oxygenate both chronically and acutely hypoxic cells in tumours. However, results have varied markedly with both tumour model and measurement technique. The current objectives were (1) to determine whether changes in radiosensitivity following oxygen manipulation correlated with changes in tumour oxygenation and (2) to assess whether oxygenation was preferentially improved in specific tumour microregions. Using two murine tumour lines, the SCCVII carcinoma and the KHT sarcoma, tumour intravascular $HbO_2$ saturations were measured cryospectrophotometrically following nicotinamide, carbogen or the combination. Generally, nicotinamide had minor effects on oxygenation, arguing against a substantial effect on acute hypoxia, while carbogen and the combination produced marked and equivalent improvements in oxygen availability. These results demonstrate that changes in tumour radiosensitivity may not agree with corresponding changes in oxygenation, even within a given tumour model, and that the efficacy of a given manipulative agent may vary substantially with tumour line. One possible explanation for these findings is that different subpopulations of clonogenic *vs* non-clonogenic cells may be oxygenated by alternative treatments.

**Keywords:** oxygen; radiosensitivity; tumour oxygenation; hypoxia; manipulation

Over the past few decades, numerous clinical and experimental studies have attempted to improve tumour radioresponse through the enhancement of tumour oxygenation. Several recent reports have indicated that either carbogen breathing (95% oxygen, 5% carbon dioxide) or nicotinamide (NIC) administration, alone or in combination, can effectively radiosensitise tumours in mice (Rojas, 1991; Chaplin *et al.*, 1993; Simon *et al.*, 1993; Siemann *et al.*, 1994; Dorie *et al.*, 1994; Martin *et al.*, 1994). It is generally believed that carbogen breathing improves response by increasing the amount of oxygen physically dissolved in the blood, thereby increasing the distance the oxygen is able to diffuse from the blood vessels to the tumour cells, and possibly by increasing tumour blood flow (Kruuv *et al.*, 1967). NIC, on the other hand, has been suggested to increase tumour oxygenation by reducing temporal fluctuations in tumour blood flow (Chaplin *et al.*, 1990, 1991; Horsman *et al.*, 1990) in addition to increasing tumour blood flow (Horsman *et al.*, 1989; Stone *et al.*, 1992; Kelleher and Vaupel, 1993).

Although previous studies have generally shown the greatest enhancement of radiosensitivity following the combination of NIC and carbogen, results are highly variable among different tumour models and laboratories. For example, in the CaNT mouse mammary carcinoma (Kjellen *et al.*, 1991), NIC demonstrated no significant effect, while carbogen and the combination showed equivalent enhancements. In the KHT sarcoma, the treatments had similar effects when delivered under optimum conditions (Siemann *et al.*, 1994). Finally, in the SCCVII carcinoma, carbogen and NIC were equivalent, while the combination was superior (Chaplin *et al.*, 1993). Measurements of alterations in tumour oxygenation following these agents have also varied markedly with tumour line (Lee and Song, 1992; Fenton and Boyce, 1993; Kelleher and Vaupel, 1993; Horsman *et al.*, 1995; Martin *et al.*, 1994).

A key question is whether a relationship exists between direct measures of tumour oxygenation and corresponding determinations of tumour radiosensitivity. Since tumour radiosensitivity is commonly calculated from the ratio of the fraction of anoxic clonogenic tumour cells to the fraction of total clonogenic cells (Moulder and Rockwell, 1984), it follows that if substantially different proportions of clonogenic and non-clonogenic tumour cells are oxygenated by the alternative treatments, direct measures of tumour oxygenation will not correlate with changes in radiosensitivity (Fenton *et al.*, 1995). Thus, although clear relationships may be demonstrated within specific tumour lines (Rofstad *et al.*, 1988; Horsman *et al.*, 1993), attempts to define similar correlations across tumour lines have proven unsuccessful (Rofstad *et al.*, 1988; Horsman *et al.*, 1995; Martin *et al.*, 1994).

The objective of the current study was to define further the physiological mechanisms responsible for the inter-tumour differences in response to these two agents. Using two murine tumour lines, the SCCVII carcinoma and the KHT sarcoma, changes in tumour intravascular oxyhaemoglobin ($HbO_2$) profiles were quantified following NIC, carbogen or the combination. The primary aims were (1) to determine whether previously reported changes in radiosensitivity, following growth and oxygen manipulation, could be explained solely on the basis of changes in oxygen availability and (2) to assess whether oxygen availability within specific regions of the tumour, i.e. interior *vs* periphery, is preferentially improved following the different treatments.

The major advantage of using tumour intravascular $HbO_2$ saturations as an index of tumour oxygenation is that microregional heterogeneities in oxygen availability can be spatially defined with an unequalled precision. While these measurements are not as direct a gauge of tumour radiosensitivity as electrode measurements of local oxygen pressures ($pO_2$), they may better reflect localised changes in tumour blood flow as well as the ability of manipulative agents to eliminate localised regions of anoxia. Neither anaesthesia nor physical restraint of the animals is required for these measurements.

### Materials and methods

### *Mice and tumour models*

The KHT tumour (Kallman *et al.*, 1967), a sarcoma maintained *in vivo*, and the SCCVII tumour, a squamous cell carcinoma maintained by alternate *in vivo*/*in vitro* passage (Olive *et al.*, 1985), were used in all experiments. Using 6- to 8-week-old female $C_3H/HeJ$ mice (Jackson Laboratories, Bar

Correspondence: BM Fenton, Box 704, University of Rochester Medical Center, Rochester, New York 14642, USA
Received 26 September 1994; revised 15 December 1994; accepted 29 December 1994

Harbor, ME, USA), $2 \times 10^5$ KHT cells were inoculated intramuscularly into the hind limb or subcutaneously into the flank. For the SCCVII tumours, $2 \times 10^5$ cells were inoculated intramuscularly into the hind limb. Tumours were selected for cyrospectrophotometric analysis when they reached a volume of between 180 and 900 $mm^3$.

*Drugs*

Nicotinamide (Sigma, St Louis, MO, USA) was freshly prepared before each experiment in sterile phosphate-buffered saline and injected intraperitoneally at 1000 mg $kg^{-1}$ 1 hr before tumour freezing.

*Carbogen breathing*

Mice were confined to plastic jigs (~50 $cm^3$ volume) and exposed to carbogen at a flow rate of ~4 l $min^{-1}$ for 7 min before tumour freezing.

*Tumour freezing and cryospectrophotometric determination of $HbO_2$ saturations*

Approximately 2–3 h before freezing, tumours were first shaved and a depilatory agent applied to accelerate tumour freezing. Following each treatment, the mice were cervically dislocated and the tumours immediately quick frozen using a liquid nitrogen-cooled copper block and stored in cryotanks. Tumour sectioning and sampling procedures were as previously described (Fenton and Boyce, 1993). Four cross-sections of the tumour were exposed using a cooled scalpel blade in a dry ice–ethanol bath at −73°C. For $HbO_2$ determinations, the exposed tumour surfaces were analysed on a liquid nitrogen-cooled microscope stage. Approximately 95 blood vessels (diameter ⩾ 6 μm) were systematically selected per tumour, the spatial positions of the blood vessels were recorded using stage micrometers and intravascular $HbO_2$ saturations were determined cryospectrophotometrically as previously described (Fenton and Gayeski, 1990). Briefly, $HbO_2$ saturations were calibrated as a function of reflected light intensity at three discrete wavelengths, based on the spectral differences between oxy- and deoxyhaemoglobin. Since optical density also varies as a function of haemoglobin concentration and light pathlength, the intensities at the three wavelengths were combined to normalise the measurement and to cancel out these dependencies, thus allowing vessels of widely varying haematocrit to be analysed using a single calibration curve.

*Statistical considerations*

The percentage of vessels containing ⩾ 25% $HbO_2$ was calculated for each of the tumours of a given treatment group, and the means at each distance class were compared using the unpaired Student *t*-test. Differences were considered significant for $P < 0.05$.

### Results

Tumour intravascular $HbO_2$ saturations vary as a function of both tumour size and spatial location within the tumour volume. In the figures to follow, results are presented in terms of the percentage of vessels ⩾ 25% $HbO_2$ saturation. Although this is a somewhat arbitrary $HbO_2$ cut-off, this type of index is expected to correlate more closely with corresponding changes in the radiobiological hypoxic fraction than mean or median $HbO_2$ levels, as has been discussed in a previous theoretical study (Fenton *et al.*, 1995).

Figure 1 illustrates the percentage of vessels with ⩾ 25% $HbO_2$ saturation as a function of distance of the vessels from the tumour surface for small (mean = 315 $mm^3$) and medium (mean = 733 $mm^3$) volume KHT tumours. The percentage with ⩾ 25% $HbO_2$ was significantly lower for the medium tumours than for the small tumours for the first two distance classes ($P = 0.002$ and $P = <0.001$). At distances of greater than 2 mm from the tumour surface, however, the percentage with ⩾ 25% $HbO_2$ was not significantly different between small and medium tumours. Figure 2 compares percentage with ⩾ 25% $HbO_2$ for KHT tumours implanted into two different sites. Tumour oxygen availability was significantly lower for the subcutaneous implantation site in the flank in relation to the intramuscular site in the hind leg at three of the four distance classes ($P = 0.002$, 0.007, 0.122 and 0.039).

Changes in the percentage with ⩾ 25% $HbO_2$ following NIC administration, carbogen breathing or the combination treatment compared with the untreated volume-matched KHT controls are summarised in Figure 3. The values obtained in NIC-treated animals were significantly different from those in untreated controls at only one of the four distance classes ($P = 0.962$, 0.018, 0.067 and 0.729). For carbogen breathing, the percentage with ⩾ 25% $HbO_2$ was significantly higher than untreated at each of the first three distance classes ($P = 0.003$, 0.006, 0.003 and 0.299). For the combination treatment, the percentage with ⩾ 25% $HbO_2$ was significantly higher at all distances ($P = 0.005$, 0.0003, 0.0005 and 0.022). Finally, the combination treatment was significantly higher than the NIC at two of the distances ($P = 0.023$, 0.163, 0.093 and 0.010) while not significantly different from the carbogen-breathing treatment at any distance.

**Figure 1** Percentage of vessels with ⩾ 25% $HbO_2$ (mean ± s.e.m.) as a function of distance from the tumour surface. Small-volume KHT tumours (■, 315 ± 46 $mm^3$, $n = 6$) are contrasted with medium volume tumours (∇, 733 ± 42 $mm^3$, $n = 6$).

**Figure 2** Percentage of vessels with ⩾ 25% $HbO_2$ (mean ± s.e.m.) as a function of distance from the tumour surface. Intramuscular KHT tumour implantations (∇, 529 ± 58 $mm^3$, $n = 8$) are contrasted with subcutaneous implantations (●, 591 ± 40 $mm^3$, $n = 7$).

Corresponding results for the SCCVII tumours are shown in Figure 4. For the SCCVII tumours, NIC produced a significant increase in percentage with $\geqslant 25\%$ $HbO_2$ only at the most peripheral distance class ($P = 0.019$). For carbogen breathing, the percentage with $\geqslant 25\%$ $HbO_2$ was again significantly increased for each of the first three distance classes ($P = 0.0001$, 0.0004, 0.0042 and 0.264). For the combination treatment, percentage with $\geqslant 25\%$ $HbO_2$ was significantly higher only for the first two distance classes ($P = 0.003$, 0.013, 0.060 and 0.842). As with the KHT, no significant differences were found between the carbogen breathing and the combination treatment. In contrast to the KHT, however, no significant differences were observed between the NIC and the combination treatment for the SCCVII tumours.

To illustrate more clearly overall variations between the two tumour lines, Figure 5 presents the mean percentage with $\geqslant 25\%$ $HbO_2$ averaged over the four distance classes. Overall the percentage of vessels with $\geqslant 25\%$ $HbO_2$ was calculated by taking the mean percentage with $\geqslant 25\%$ $HbO_2$ at each distance class, weighted by the corresponding tumour volume associated with that distance class for each tumour. This weighting compensates for the fact that the inner distance classes sample from smaller concentric shells of the tumour volume than at the outer distance classes. Trends for the KHT and SCCVII tumours generally parallel the previous figures. For the KHT, significant overall differences from untreated were found only for the carbogen and combination treatments ($P = 0.006$ and 0.0008 respectively). Carbogen and the combination were also significantly higher than NIC alone ($P = 0.080$ and 0.020). For the SCCVII, carbogen and the combination were again significantly higher than untreated ($P = 0.0003$ and 0.013), NIC was different from carbogen ($P = 0.015$) but not the combination ($P = 0.206$).

**Figure 3** Percentage of vessels with $\geqslant 25\%$ $HbO_2$ (mean ± s.e.m.) as a function of distance from the tumour surface for KHT i.m. implanted tumours. Untreated controls (▽, $529 \pm 58$ $mm^3$, $n = 8$) are contrasted with NIC administration (▼, $530 \pm 65$ $mm^3$, $n = 9$), carbogen breathing (●, $483 \pm 27$ $mm^3$, $n = 7$) or the combination of both (■, $430 \pm 25$ $mm^3$, $n = 7$).

**Figure 4** Percentage of vessels with $\geqslant 25\%$ $HbO_2$ (mean ± s.e.m.) as a function of distance from the tumour surface for SCCVII i.m. implanted tumours. Untreated controls (▽, $503 \pm 62$ $mm^3$, $n = 8$) are contrasted with NIC administration (▼, $456 \pm 43$ $mm^3$, $n = 7$), carbogen breathing (●, $423 \pm 30$ $mm^3$, $n = 7$) or the combination of both (■, $460 \pm 18$ $mm^3$, $n = 7$).

### Discussion

As a rule, the radiobiological hypoxic fraction (HF) of experimental tumours increases with increasing tumour volume within a given tumour line (Rofstad *et al.*, 1988; Horsman *et al.*, 1995). For KHT tumours grown in the leg muscle, the HF increased from ~10% in 0.1–0.2 g tumours to 25–35% for 0.7–1.0 g tumours (Fenton and Siemann, 1994). As the HFs increased, the corresponding percentage with $\geqslant 25\%$ $HbO_2$ decreased, as expected. But this decrease in tumour oxygen availability was not uniformly distributed over the tumour volume. Significant changes in tumour oxygenation were confined to blood vessels within 2 mm of the tumour surface. For vessels closer to the centre of the tumour, $HbO_2$ levels were quite low to begin with and were not significantly decreased with growth.

$HbO_2$ levels also varied substantially with implantation site. Although the radiobiological HFs of leg- and flank-implanted KHT tumours were similar (Fenton and Siemann, 1994), the percentage with $\geqslant 25\%$ $HbO_2$ for the leg tumours was substantially higher. Despite essentially equal HFs for flank or leg KHT tumours, Horsman *et al.* (1995) also found 2–4 times higher median $pO_2$ values for leg than for flank tumours. This increase in oxygen availability in the leg tumours may relate to differences in the host vasculature or relative blood flow in the different sites, among other factors (Young *et al.*, 1979; Vaupel and Mueller-Klieser, 1986). Since the leg muscle would be expected to have higher oxygen requirements than the subcutaneous flank, it stands to reason that leg tumours should also tend to be better vascularised and oxygenated. It remains difficult to explain why the $HbO_2$ or $pO_2$ levels do not correlate with the hypoxic fractions between the two sites, although, if the tumour cells surrounding the low-$HbO_2$ vessels in the flank are predominantly non-clonogenic, the resultant oxygen profiles would be lower in this site than in the leg without a corresponding increase in the radiobiological hypoxic fraction (Fenton *et al.*, 1995).

**Figure 5** Overall percentage vessels with $\geqslant 25\%$ $HbO_2$ saturation (mean ± s.e.m.) as a function of treatment for KHT (■) and SCCVII (□) tumours. 'Both' denotes the combination of NIC and carbogen, and numbers of tumours are as described for Figures 4 and 5.

A number of additional mechanisms have also been suggested previously (Rofstad *et al.*, 1988).

Carbogen breathing and the combination of carbogen plus NIC provided the greatest enhancement of tumour oxygen availability for both the KHT and the SCCVII tumours, and this enhancement was not significantly different between the two treatments. $HbO_2$ levels were improved for only one distance class following NIC for either KHT or SCCVII tumours. Although these results suggest that tumour radiosensitivity should also tend to be highest following the carbogen and combination treatments, published changes in radioresponse following these agents do not support this conclusion. Siemann *et al.* (1994) found that NIC and carbogen were equally effective at radiosensitising KHT tumours. In addition, the radiosensitivity enhancement resulting from the combination of NIC and carbogen was equivalent to either agent alone if delivered under optimum conditions. These findings differ from the current results primarily in respect of the effect of NIC administration, in that tumour radiosensitivity is increased without a marked change in tumour oxygenation.

For subcutaneous SCCVII tumours, Chaplin *et al.* (1993) also reported that carbogen and NIC produced equivalent improvements in radioresponse. In contrast to the KHT tumours, however, the combination treatment produced a greater enhancement of radiation response than either agent alone. The radiosensitisation following either NIC or carbogen was essentially the same, despite markedly different $HbO_2$ profiles. Since the combination treatment produced the same effect on radiosensitivity as the fully aerobic response in the SCCVII tumours (Chaplin *et al.*, 1993), and since the interior of these tumours remains very poorly oxygenated following the combination treatment, it follows that these interior tumour cells must be non-clonogenic to begin with and therefore irrelevant in terms of radiotherapy.

In a study using C3H mouse mammary carcinomas (Horsman *et al.*, 1995), changes in the fraction of $pO_2$ readings $\leqslant 5$ mmHg generally correlated with the corresponding HFs following different oxygen manipulations. However, changes in tumour $pO_2$ levels following NIC and carbogen were again inconsistent. Tumours were much better oxygenated following carbogen than NIC, in spite of equivalent radiosensitivities following either treatment. As was the case for the $HbO_2$ measurements, $pO_2$ levels following NIC were not different from the air-breathing controls. In the studies of Martin *et al.* (1994), the relationship between $pO_2$ profiles and surviving fraction varied markedly among tumour lines. In one, cell survival remained essentially constant between NIC and carbogen treatments, in spite of substantial differences in $pO_2$ profiles. Surprisingly, NIC increased tumour oxygenation more than carbogen in all three lines. Additional studies have shown similar variability between tumour lines in the radioresponse following NIC, carbogen or the combination (Kjellen *et al.*, 1991; Simon *et al.*, 1993; Dorie *et al.*, 1994).

The current disparity between $HbO_2$ results and HF changes can be rationalised if it is assumed that NIC and carbogen oxygenate different subpopulations of clonogenic *vs* non-clonogenic tumour cells. While direct measures of tumour oxygenation cannot distinguish between clonogenic and non-clonogenic cells, the radiobiological HF depends directly on the relative fractions of anoxic and oxygenated clonogenic cells contained in the tumour (Moulder and Rockwell, 1984; Fenton *et al.*, 1995). As described more fully in a previous theoretical study (Fenton *et al.*, 1995), HF determinations can vary independently of directly measured changes in tumour oxygenation within the non-clonogenic subpopulation. Thus, if a higher proportion of non-clonogenic *vs* clonogenic cells is oxygenated following a given treatment, higher oxygen levels will be observed in relation to the corresponding reduction in cell survival.

Carbogen breathing is believed to improve tumour oxygenation primarily by increasing the diffusion distance of the oxygen from the blood vessels – thus the clonogenic anoxic cells at the edge of the previously oxygenated regions will be the first cells oxygenated. As the oxygen diffuses further, anoxic cells may be reached that have been without oxygen long enough to become non-clonogenic while remaining viable. Increasing the diffusion distance enough to oxygenate these non-clonogenic cells results in an 'overkill' phenomenon in which no further enhancement of tumour radiosensitivity is realised in spite of the increased oxygen availability. Since the HF decreases in conjunction with the increase in tumour oxygenation following carbogen, an increase in oxygen delivery to some proportion of the clonogenic anoxic cells must also be occurring.

Previous work has suggested that NIC may act in part by reducing intermittent fluctuations in tumour blood flow (Chaplin *et al.*, 1990). If this is the case, some of the tumour cells that are oxygenated following NIC will be those that were previously exposed to intermittent flow. It is reasonable that such acutely hypoxic cells would more likely remain clonogenic than cells that have been beyond the diffusion distance of oxygen for extended periods of time (chronic hypoxia). This implies that differences in the frequency of intermittent blood flow between tumour lines could directly influence the correlation between tumour oxygenation and radioresponse for these same tumour lines. Other evidence that NIC may act by reducing intermittent flow is provided by Lee and Song (1992), who found that the effect of NIC administration was greater in large tumours than in small. They also attribute these differences to the fact that larger tumours are more likely to have intermittently opening blood vessels than small tumours (Chaplin *et al.*, 1986; Trotter *et al.*, 1989; Lord *et al.*, 1993).

But do such differences in intermittent flow exist between the KHT and SCCVII tumour lines? For SCCVII tumours, the number of blood vessels opening and closing over a 20 min period has been reported to be 10.3% (Chaplin *et al.*, 1990), based on dual-staining techniques. In the KHT tumours, only 4% intermittently flowing vessels were observed (Fenton and Siemann, 1994). Thus, in either case, a reduction in intermittent flow may have a relatively minor overall effect on tumour oxygenation. Although NIC-induced improvements in $HbO_2$ levels were observed only in the peripheral vessels, Chaplin *et al* (1990) report that flow intermittencies are, in contrast, more prevalent in central tumour regions. However, their dual-staining techniques are only capable of measuring whether a given blood vessel contains active blood flow – not whether this flow is functional in terms of oxygen delivery (Fenton and Boyce, 1993). Thus changes in dual-staining intermittency for blood vessels containing very low $HbO_2$ levels may or may not relate to either tumour oxygenation or radioresponse. Since overall $HbO_2$ levels were not substantially improved following NIC for either the KHT or the SCCVII, it appears unlikely that a NIC-induced decrease in acute hypoxia is the predominant mechanism for altering radioresponse in either tumour model. This suggests that the beneficial effects of combining NIC and carbogen may not involve a decrease in acutely hypoxic cells in all cases.

Finally, why are SCCVII and KHT $HbO_2$ levels increased so much more with carbogen than with NIC, despite similar effects on radiosensitivity? One possibility, is that, although both treatments may increase oxygen delivery to the clonogenic anoxic subpopulation, the carbogen may also tend to oxygenate some population of non-clonogenic anoxic cells. Following NIC, radioresponse increases with a minimal increase in oxygen availability, suggesting a redistribution of oxygen from non-clonogenic oxygenated cells to clonogenic anoxic cells. If NIC also reduces intermittent flow, then oxygen that was previously distributed to the non-clonogenic cells most distant from the previously open vessels will now be diverted to the clonogenic cells surrounding the newly opened vessels. Thus, less flow is now distributed among more vessels. This tends to decrease the oxygen diffusion distance while increasing oxygen delivery to the closest (and presumably clonogenic) tumour cells.

A final possible explanation for the NIC-induced radiosensitisation in the absence of significantly higher oxygen levels is the possibility that NIC may act by inhibiting radiation-induced potentially lethal damage repair. While this result has been demonstrated *in vitro*, further studies have suggested that repair inhibition is not the principle mechanism responsible for *in vivo* tumour radiosensitisation (Horsman *et al.*, 1987).

In summary, it is clear that response to carbogen and NIC manipulation varies substantially with tumour line in terms of both tumour radiosensitivity and direct measures of tumour oxygenation. The dilemma is that no currently available method exists for predicting whether or not a correlation will exist between the two measures in a given tumour. Thus defining an 'optimal' manipulative agent solely on the basis of its ability to increase tumour oxygenation may lead to erroneous conclusions. Contrary to some previous findings (Martin *et al.*, 1994), alterations in tumour oxygenation within a given tumour line may not be reflective of corresponding changes in tumour radiosensitivity if significantly different fractions of non-clonogenic tumour cells are involved. Further work is needed both to describe the underlying physiological basis for the observed discrepancies and to discover more representative methods for estimating tumour radioresponse following oxygen manipulation. In addition, better methods for quantifying intermittencies in 'functional flow' are needed such that more subtle changes in tumour perfusion may be recognised.

**Acknowledgements**

The author would like to thank Dr Thomas EJ Gayeski, Department of Anesthesiology, University of Rochester Medical Center, for the use of his laboratory and cryospectrophotometer, Deborah Boyce and Michelle Egan for excellent technical assistance, and Dr Richard Raubertas, Department of Biostatistics, for statistical assistance. Financial support was provided by NIH Grants CA52586, CA55300 and HL03290.

**References**

CHAPLIN DJ, DURAND RE AND OLIVE PL. (1986). Acute hypoxia in tumors: implications for modifiers of radiation effects. *Int. J. Radiat. Oncol. Biol. Phys.*, **12**, 1279-1282.

CHAPLIN DJ, HORSMAN MR AND TROTTER MJ. (1990). Effect of nicotinamide on the microregional heterogeneity of oxygen delivery within a murine tumor. *J. Natl Cancer Inst.*, **82**, 672-676.

CHAPLIN DJ, HORSMAN MR AND AOKI DS. (1991). Nicotinamide, fluosol DA and carbogen: a strategy to reoxygenate acutely and chronically hypoxic cells *in vivo*. *Br. J. Cancer*, **63**, 109-113.

CHAPLIN DJ, HORSMAN MR AND SIEMANN DW. (1993). Further evaluation of nicotinamide and carbogen as a strategy to reoxygenate hypoxic cells *in vivo*: importance of nicotinamide dose and pre-irradiation breathing time. *Br. J. Cancer*, **68**, 269-273.

DORIE MJ, MENKE D AND BROWN JM. (1994). Comparison of the enhancement of tumor responses to fractionated irradiation by SR 4233 (tirapazamine) and by nicotinamide with carbogen. *Int. J. Radiat. Oncol. Biol. Phys.*, **28**, 145-150.

FENTON BM AND BOYCE DJ. (1993). Micro-regional mapping of $HbO_2$ saturations and blood flow following nicotinamide administration. *Int. J. Radiat. Oncol. Biol. Phys.*, **29**, 459-462.

FENTON BM AND GAYESKI TEJ. (1990). Determination of microvascular oxyhemoglobin saturations using cryospectrophotometry. *Am. J. Physiol.*, **259**, H1912-H1920.

FENTON BM AND SIEMANN DW. (1994). Investigations of perfusion-limited hypoxia and oxygenation in the KHT sarcoma. In *Oxygen Transport to Tissue, Vol. XVI*, Hogan MC, Mathieu-Costello O and Wagner PD (eds) pp. 623-630. Plenum Press: New York.

FENTON BM, KIANI MF AND SIEMANN DW. (1995). Should direct measurements of tumor oxygenation relate to the radiobiological hypoxic fraction of a tumor? *Int. J. Radiat. Oncol. Biol. Phys.* (in press).

HORSMAN MR, CHAPLIN DJ AND BROWN JM. (1987). Radiosensitization by nicotinamide *in vivo*: a greater enhancement of tumor damage compared to that of normal tissues. *Radiat. Res.*, **109**, 479-489.

HORSMAN MR, CHAPLIN DJ AND BROWN JM. (1989). Tumor radiosensitization by nicotinamide: a result of improved perfusion and oxygenation. *Radiat. Res.*, **118**, 139-150.

HORSMAN MR, CHAPLIN DJ AND OVERGAARD J. (1990). Combination of nicotinamide and hyperthermia to eliminate radioresistant chronically and acutely hypoxic tumor cells. *Cancer Res.*, **50**, 7430-7436.

HORSMAN MR, KHALIL AA, NORDSMARK M, GRAU C AND OVERGAARD J. (1993). Relationship between radiobiological hypoxia and direct estimates of tumour oxygenation in a mouse tumour model. *Radiother. Oncol.*, **28**, 69-71.

HORSMAN MR, KHALIL AA, NORDSMARK M, SIEMANN DW, HILL SA, LYNCH EM, CHAPLIN DJ, STERN S, THOMAS CD, GUICHARD M, GRAU C AND OVERGAARD J. (1994). The use of oxygen electrodes to predict radiobiological hypoxia in tumours. In *Tumor Oxygenation*, Vaupel P, Kelleher DK and Guenderoth M (eds) Gustav Fischer: Stuttgart.

KALLMAN RF, SILINI G AND VAN PUTTEN LM. (1967). Factors influencing the quantitative estimation of the in vivo survival of cells from solid tumors. *J. Natl Cancer Inst.*, **39**, 539-549.

KELLEHER DK AND VAUPEL PW. (1993). Nicotinamide exerts different acute effects on microcirculatory function and tissue oxygenation in rat tumors. *Int. J. Radiat. Oncol. Biol. Phys.*, **26**, 95-102.

KJELLEN E, JOINER MC, COLLIER JM, JOHNS H AND ROJAS A. (1991). A therapeutic benefit from combining normobaric carbogen or oxygen with nicotinamide in fractionated X-ray treatments. *Radiother. Oncol.*, **22**, 81-91.

KRUUV JA, INCH WR AND McCREDIE JA. (1967). Blood flow and oxygenation of tumors in mice. I. Effects of breathing gases containing carbon dioxide at atmospheric pressure. *Cancer Res.*, **20**, 51-59.

LEE I AND SONG CW. (1992). The oxygenation of murine tumor isografts and human tumor xenografts by nicotinamide. *Radiat. Res.*, **130**, 65-71.

LORD EM, HARWELL L AND KOCH CJ. (1993). Detection of hypoxic cells by monoclonal antibody recognizing 2-nitroimidazole adducts. *Cancer Res.*, **53**, 5721-5726.

MARTIN LM, THOMAS CD AND GUICHARD M. (1994). Nicotinamide and carbogen: relationship between pO2 and radiosensitivity in three tumour lines. *Int. J. Radiat. Biol.*, **65**, 379-386.

MOULDER JE AND ROCKWELL S. (1984). Hypoxic fraction of solid tumors: Experimental techniques, methods of analysis, and a survey of existing data. *Int. J. Radiat. Oncol. Biol. Phys.*, **10**, 695-712.

OLIVE PL, CHAPLIN DJ AND DURAND RE. (1985). Pharmacokinetics, binding, and distribution of Hoechst 33342 in spheroids and murine tumors. *Br. J. Cancer*, **52**, 739-746.

ROFSTAD EK, FENTON BM AND SUTHERLAND RM. (1988). Intracapillary $HbO_2$ saturations in murine tumours and human tumour xenografts measured by cryospectrophotometry: relationship to tumour volume, tumour pH and fraction of radiobiologically hypoxic cells. *Br. J. Cancer*, **57**, 494-502.

ROJAS A. (1991). Radiosensitization with normobaric oxygen and carbogen. *Radiother. Oncol.*, **20**, 65-70.

SIEMANN DW, HORSMAN MR AND CHAPLIN DJ. (1994). The radiation response of KHT sarcomas following nicotinamide treatment and carbogen breathing. *Radiother. Oncol.*, **31**, 117-122.

SIMON JM, LARTIGAU E AND GUICHARD M. (1993). Nicotinamide and carbogen: major effect on the radiosensitivity of EMT6 and HRT18 tumours. *Radiother. Oncol.*, **28**, 203-207.

STONE HB, MINCHINTON AI, LEMMON M, MENKE D AND BROWN JM. (1992). Pharmacological modification of tumor blood flow: lack of correlation between alteration of mean arterial blood pressure and changes in tumor perfusion. *Int. J. Radiat. Oncol. Biol. Phys.*, **22**, 79-86.

TROTTER MJ, CHAPLIN DJ, DURAND RE AND OLIVE PL. (1989). The use of fluorescent probes to identify regions of transient perfusion in murine tumors. *Int. J. Radiat. Oncol. Biol. Phys.*, **16**, 931-934.

VAUPEL P AND MUELLER-KLIESER W. (1986). Cell line and growth site as relevant parameters governing tumor tissue oxygenation. *Adv. Exp. Med. Biol.*, **200**, 633-643.

YOUNG SW, HOLLENBERG NK AND ABRAMS, HL. (1979). The influence of implantation site on tumor growth and blood flow. *Eur. J. Cancer*, **15**, 771-777.